# Molecular Identification of *Babesia* spp. and *Anaplasma marginale* in Water Buffaloes in Veracruz and Tabasco, Mexico: A Retrospective Study

**DOI:** 10.3390/microorganisms10091702

**Published:** 2022-08-24

**Authors:** José Juan Lira-Amaya, Rebeca Montserrat Santamaria-Espinosa, Roberto O. Castañeda-Arriola, Grecia Martínez-García, Diego J. Polanco-Martínez, Carmen Rojas-Martínez, Jesús Ántonio Alvarez-Martínez, Julio V. Figueroa-Millán

**Affiliations:** 1 CENID-Salud Animal e Inocuidad, Instituto Nacional de Investigaciones Forestales, Agrícolas y Pecuarias, Carretera Federal Cuernavaca Cuautla No. 8534, Colonia Progreso, Jiutepec 62550, Mexico; 2 Sitio Experimental Pichucalco, CIRGOC, Instituto Nacional de Investigaciones Forestales, Agrícolas y Pecuarias, Km. 8, Carretera Pichucalco-Teapa, Pichucalco 86040, Mexico

**Keywords:** PCR, *Babesia*, *Anaplasma*, buffaloes, DNA sequencing, BLASTn

## Abstract

Two hundred and thirty-three blood samples of water buffalo were collected on four farms in Veracruz state and Tabasco state, Mexico, to detect and confirm the identities of *Babesia* and *Anaplasma* spp. sequences. Nested PCR assays were used for the amplification of specific genes encoding *B. bovis* rhoptry-associated protein (RAP-1), *B. bigemina Spe*I-*Ava*I restriction fragment, and *Anaplasma marginale* major surface protein 5 (MSP5). Using DNA sequencing and BLASTn analysis for DNA homology hemoparasite identification, the identities of the hemoparasites were established by comparing the nucleotide sequences obtained in this study with those available in the GenBank database at the National Center for Biotechnology Information (NCBI). Water buffalo infection with at least one of the hemoparasites under study was detected in 45% (105/233) of the blood samples, while a mixed infection with *B. bovis* and *B. bigemina* was detected in 6.4% (15/233) of samples. For this cross-sectional study, mixed infections with the three hemoparasites were not detected. BLASTn analysis revealed that the nucleotide sequences of the water buffalo isolates shared sequence identity values ranging from 88 to 100% with previously published gene sequences of *B. bovis*, *B. bigemina*, and *A. marginale*. The current results confirm that water buffalo, as cattle, are also carriers of hemoparasite infections that are tick-transmitted, and suggest that they probably have an important role in the epidemiology of bovine babesiosis in Mexico.

## 1. Introduction

More than 35 million cattle are recorded in Mexico [1], of which 70% are located in tropical and subtropical regions, geographical areas that favor the proliferation of different etiological agents causing diseases of importance for animal health [2]. In tropical livestock production facilities, 19.5% of milk and 40% of the meat consumed in the country are produced predominantly using the dual-purpose system of cattle production units [3]; however, the appearance of certain infectious diseases, including tick-borne diseases, represents one of the greatest drawbacks for the full development of national livestock, due to the economic losses these diseases impose on the cattle industry [4]. Ticks can transmit to both domestic and wild animals a greater variety of pathogens than any other group of vectors, including the intra-erythrocytic parasites *Babesia bovis* and *Babesia bigemina*, etiologic agents of bovine babesiosis [5,6]. Bovine babesiosis is an infectious disease that is widely distributed in tropical regions around the world. Due to the high rate of morbidity and mortality in cattle, the most important species are *Babesia bovis* and *Babesia bigemina* [7,8]. Both protozoan species are present in Mexico and are transmitted by *Rhipicephalus microplus* and *Rhipicephalus annulatus* ticks [9].

Bovine anaplasmosis is another disease of great importance that is transmitted by vectors and that also conditions the subsistence of livestock production. The disease can be caused by *Anaplasma marginale* and *Anaplasma centrale*, but only *A. marginale* has been reported in Mexico [10]. Babesiosis and anaplasmosis not only affect domestic cattle [11], but these diseases have also been described in wild cattle, such as water buffaloes and American bison [12,13,14].

Immunological tests for the detection of circulating antibodies in bovine serum have been previously used in epidemiological studies in Mexico [9,15]. However, more sensitive and specific diagnostic alternatives for the detection and control of these diseases are the use of nucleic acid probes and the polymerase chain reaction (PCR) test [16,17,18]. Due to their high production potential in tropical regions, water buffaloes (*Bubalus bubalis*) were introduced in Mexico as an alternative in addition to livestock production more than two decades ago. This species of wild animals, as well as bovines, are susceptible to several microorganisms [19,20]. However, in Mexico, there is scarce scientific information that can corroborate infection with hemoparasites in water buffaloes, such as those that cause bovine babesiosis and anaplasmosis, as has been reported in other geographical latitudes [12,13,21,22].

To confirm the identity of these hemoparasites, it is necessary to use molecular methods that allow for the determination of the specificity and degree of sequence conservation for the target diagnostic genes. Therefore, the main objective of the present study is to detect, with PCR assays and confirm by DNA sequencing of amplified target genes, the presence of *Babesia* and *Anaplasma* sp. in samples from infected water buffaloes in Veracruz and Tabasco, Mexico.

## 2. Materials and Methods

**Ethical considerations.** The study was approved by the Animal Experimentation and Ethics Committee of the National Center for Disciplinary Research in Animal Health and Safety (CENID-SAI, INIFAP, Jiutepec, Mexico). The study took into consideration the ethical and methodological aspects, in accordance with Mexican regulations on the use, housing, and transport of experimental animals (NOM-062-ZOO-1999 and NOM-051-ZOO-1995).

**Design and study population**. A cross-sectional study was conducted in 2017, using a non-probabilistic sampling method for convenience. The total population of water buffaloes from two production units (PU), located at the municipality of Sayula de Aleman, state of Veracruz, Mexico, were sampled for this study: Veracruz 1 (*n* = 61) and Veracruz 2 (*n* = 63). The altitude of the region where the study was carried out is 80 m above sea level. The climate in the region is warm sub-humid, with mean temperatures ranging between 24 and 28 °C. Similarly, the total population of water buffaloes from two production units (PU), in the municipality of Villahermosa, Tabasco state, Mexico, was sampled for this study: Tabasco 1 (*n* = 58) and Tabasco 2 (*n* = 51), for a grand total of 233 water buffalo samples. Blood samples were collected using vacuum tubes containing anticoagulant (EDTA) through the puncture of the coccygeal vein. The samples were processed using tube centrifugation at 3500 rpm for 5 min. After removing the buffy coat, the erythrocyte pellets were kept in refrigeration at 4 °C until their arrival at the laboratory (CENID-SAI, INIFAP in Jiutepec, Mexico), where they remained stored at −20 °C until DNA extraction in this study.

**DNA extraction and detection by nested PCR test**. Genomic DNA extraction was performed from the erythrocyte pellets using a commercial kit (MO BIO Laboratories, Carlsbad, CA, USA), following the manufacturer’s instructions. For molecular detection via nested PCR (nPCR) assays, primers designed to amplify specific genes that encode for the rhoptry-associated protein (*rap*-1), the restriction fragment *Spe*I-*Ava*I, and the main surface protein 5 (MSP5) of *B. bovis*, *B. bigemina*, and *A. marginale*, respectively, were used as described (Table 1) [16,17,18]. It has been indicated that the nested PCR assay is notoriously sensitive to false positives. In this study, the procedures of good laboratory practice were followed to avoid contamination in each step during the whole experiment [23].

**Cloning and sequencing**. Three nPCR-positive samples of *B. bovis*, *B. bigemina*, and *A. marginale* for each PU were randomly selected for genetic characterization, except for PU Veracruz 1 and Veracruz 2, where only one and two positive samples of *A. marginale* and *B. bigemina* were found, respectively. The PCR-amplified DNA fragments were inserted in plasmid vector PCR 2.1 TOPO (Invitrogen, Carlsbad, CA, USA), and transformed in chemically competent TOP10 E. coli host cells [24]. Subsequently, two white (recombinant) colonies selected for each of the samples were proliferated in liquid LB medium with ampicillin (50 μg/mL). Plasmid DNA was purified with a commercial kit (PROMEGA, Madison, WI, USA) and was submitted to the Biotechnology Institute, UNAM, Mexico, as a template for DNA sequencing by using the Sanger method with fluorolabeled dideoxynucleotides as chain terminators. Nucleotide sequences were used to establish the identity of hemoparasite DNA by aligning the sequences with the GenBank database of the National Center for Biotechnology Information (NCBI), using the BLASTn program available online (https://blast.ncbi.nlm.nih.gov/Blast.cgi, accessed on 17 June 2022).

## 3. Results

**Figure 1:**
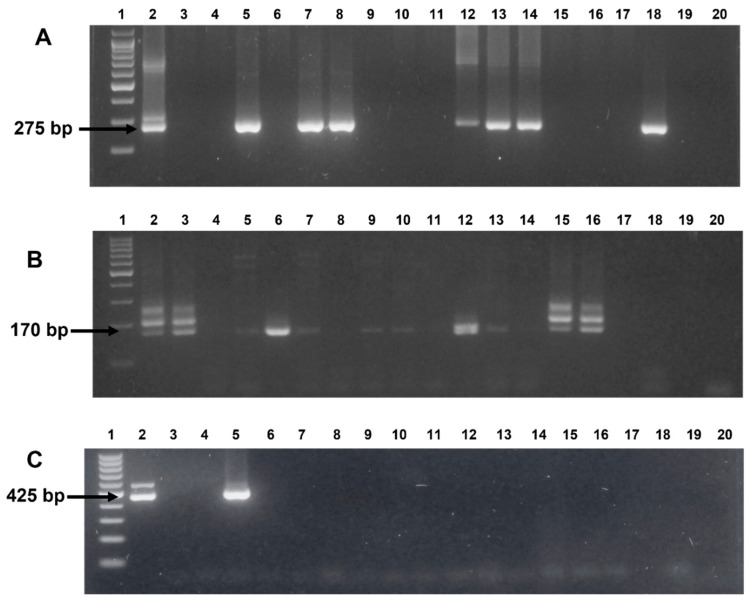
Representative image of nested PCR-amplified products using primers specific for (**A**) ***Babesia bovis*.** Lanes: 1, molecular marker 100 bp; 2, Positive bovine control; 3, Negative bovine control; 4–18, Buffalo samples; 19–20, Nuclease-free water control. (**B**) ***Babesia bigemina*.** Lanes: 1, molecular marker 100 bp; 2–3, Positive bovine controls; 4–16, Buffalo samples; 17, Negative bovine control; 18 and 20, Nuclease-free water control; 19, empty. (**C**) ***Anaplasma marginale***. Lanes: 1, molecular marker 100 bp; 2, Positive bovine control; 3, Negative bovine control; 4, Nuclease-free water control; 5–17, Buffalo samples; 18, empty; 19–20, Nuclease-free water controls.

A total of 233 blood samples were tested using the nPCR assay for infection with *B. bovis*, *B. bigemina*, and *A. marginale* in water buffaloes. In Figure 1, a picture of ethidium bromide-stained agarose gels is presented, showing the representative results of the nPCR assays conducted for all three pathogens searched for in this study. Importantly, the image shows the results obtained with nPCR assays run on DNA extracted either from uninfected bovine erythrocytes (negative control), or bovine erythrocytes infected with *B. bovis*, *B. bigemina*, or *A. marginale* (positive control).

Infection with at least one of the hemoparasites was detected in 45% (105/233) of the samples, while in 6.4% (15/233) of the samples, a mixed infection with *B. bovis* and *B. bigemina* was detected.

In the PU Veracruz 1, DNA from *B. bovis* and *B. bigemina* was detected in 37.7% (23/61) and 9.8% (6/61) of the samples, respectively. *A. marginale* was the least prevalent species in this PU, as it was detected in only 1.6% (1/61) of the samples (Table 2). Regarding the PU Veracruz 2, the parasite species with the highest prevalence was also *B. bovis* (20.6%) as compared to *B. bigemina* (11.1%) and *A. marginale* (6.3%), as shown in Table 2.

In contrast, in PU Tabasco 1, DNA from *B. bovis* and *B. bigemina* was detected in 60.3% (35/58) and 13.7% (8/58) of the samples, respectively. *A. marginale* was not detected in this PU (Table 2). In PU Tabasco 2, the parasite species with the highest prevalence was also *B. bovis* (41.1%), as compared to *B. bigemina* (23.5%) and *A. marginale* (1.9%), as shown in Table 2.

DNA sequences of amplified genes encoding the rhoptry-associated protein (RAP-1), the *Spe*I-*Ava*I restriction fragment, and the major surface protein 5 (MSP5) of *B. bovis*, *B. bigemina*, and *A. marginale*, respectively, were genetically characterized.

Out of the 12 PCR-purified *B. bovis rap*-1 amplicons (275 bp) that were cloned, recombinant *E. coli* colonies (three from each sample) were selected prior to DNA sequencing, by verifying the insertion in the plasmid vector of the DNA fragment of interest (275 bp). Twelve representative consensus *B. bovis rap*-1 fragment DNA sequences were assembled and submitted to GenBank, obtaining the corresponding accession numbers ON862140–ON862151 for each of the *B. bovis* isolates that were cloned and derived from the infected water buffaloes.

Similarly, out of the 12 PCR purified *B. bigemina Spe*I-*Ava*I amplicons (171 bp) that were cloned, recombinant *E. coli* colonies (three from each sample) were obtained for DNA sequencing, by verifying the insertion in the plasmid vector of the DNA fragment of interest (171 bp). Eleven representative consensus *B. bigemina Spe*I-*Ava*I fragment DNA sequences were assembled and submitted to GenBank, obtaining the corresponding accession numbers ON862152–ON862162 for each of the *B. bigemina* isolates that were cloned and derived from the infected water buffaloes.

Regarding the *msp*-5 amplicons (425 bp) derived from *A. marginale*-infected water buffaloes, only four consensus sequences were assembled and submitted to the GenBank repository. The accession numbers provided were ON862163–ON862166 for the *A. marginale* isolate DNAs that were cloned and derived from the infected water buffaloes.

BLASTn analysis of the nucleotide sequences of water buffalo samples revealed sequence identity values ranging from 88 to 100%, with reference sequences of *B. bovis*, *B. bigemina*, and *A. marginale* deposited in GenBank (Table 3). It should be mentioned that the positive control amplicons were also sequenced and submitted to the GenBank repository (accession numbers OP174624, OP174630, and OP174628 for the amplicon DNA sequences, corresponding to *B. bovis*, *B. bigemina*, and *A. marginale*, respectively). BLASTn homology search analyses of the positive control, amplicon sequences showed 100% sequence identity with the previously reported *B. bovis*, *B, bigemina*, and *A. marginale* DNA sequences, which are available in the GenBank repository.

The comparative analyses of the nucleotide sequences that were obtained in this study showed a high overall sequence identity (99–100%) between *B. bovis rap*-1 from the Mexican water buffalo isolates (Table 3) when compared with the *B. bovis rap*-1 reference sequence derived from cattle, GenBank AF030056.2 “S2P” strain [25], and more importantly, when compared to the *B. bovis rap*-1 sequences derived from water buffaloes, GenBank JF279443 “Cuba” isolate [13], and GenBank AB845432 “Polonnaruwa” isolate from Sri Lanka [26].

The DNA sequence identities of 96–99% between *B. bigemina Spe*I-*Ava*I from the Mexican water buffalo isolates (Table 3) were determined, when compared with the *B. bigemina Spe*I-*Ava*I reference sequence derived from cattle, the GenBank S45366.1 “Mexico” strain [17]. The lowest sequence identity was identified in sample No. 75.1, Accession No. ON862161, which had only 88% identity with the *Spe*I-*Ava*I sequence deposited in the GenBank as FJ939721.1 of the “Setubal” isolate from Portugal [27].

A comparative analysis of the *A. marginale msp*5 nucleotide sequences that were obtained in this study showed sequence identities from 96 to 99% between the *A. marginale msp*5 from the Mexican water buffalo isolates (Table 3), when compared with the *A. marginale msp*5 reference sequence derived from water buffalo, the GenBank LC467711.1 “Mullaitivu” isolate from Sri Lanka [28].

## 4. Discussion

By using highly sensitive molecular tests such as nPCR, the analyte concentrations of at least 100 and 0.1 fg of genomic DNA can be detected in samples containing *B. bigemina* and *B. bovis* DNA, respectively [9,16,17,18].

The diagnostic sensitivities and specificities of the PCR assays developed to detect *Babesia bovis-* and *B. bigemina*-infected blood samples have been previously reported [29]. In that study, the diagnostic efficacy of the light microscopy examination of blood smears (the standard gold standard test for the diagnosis of acute cases of cattle babesiosis) and the polymerase chain reaction assay were evaluated in terms of their ability to detect cattle that were experimentally infected with *Babesia bovis* and *Babesia bigemina* (while *Anaplasma marginale* was also evaluated, the PCR assay for this pathogen included a primer set different to that used in the present study). Blood samples were collected from 32 intact, 1–2-year-old Holstein bulls, previous to (true negative samples) and after simultaneous inoculation of culture-derived or field isolates of *B. bovis*- and *B. bigemina*-infected erythrocytes (true positive samples). The results of this study show that both tests had 100% specificity. In contrast, the sensitivities of the PCR assay against the light microscopy test were 93.5% and 70.9% for *B. bovis*, respectively, whereas 96.7% and 100% were determined in the *B. bigemina* infection, respectively. The advantages of using the PCR assay to differentially diagnose cattle with dual hemoparasite infection were clearly demonstrated. The set of primers of this study were later validated in both *Babesia*-infected ticks and bovine hosts [30], and due to its high analytical sensitivity to detect low parasitemias, it was recommended by the OIE as the nPCR standard to detect and confirm *Babesia bovis* and/or *B. bigemina* in chronically infected carrier cattle [31]. Nonetheless, the primers that target the *B. bigemina* SpeI-AvaI restriction fragment can also amplify *B. ovata* DNA [32]. Therefore, it was advised that care should be taken when testing DNA samples that are from *B. ovata*-endemic regions (Japan). While this is not the case for the samples tested in this study, we decided to perform DNA sequence analyses of some of the amplicons obtained from the buffaloes, as well as from the positive controls, to have confidence in the results.

As for the *A. marginale* detection test, nPCR assays to detect *A. marginale* infection in carrier cattle have been developed, although not yet fully validated until recently. The assay developed by Torioni de Echaide et al. (1998) [18] was cited by the OIE as one of the nPCR assays used to identify *A. marginale* carrier cattle [33]. Moreover, the sensitivity and specificity values for the blood smear detection of *A. marginale* were recently assessed, showing 85.6% and 98.8%, respectively. In contrast, the sensitivity and specificity for the MSP5-based nPCR detection of *A. marginale* was 95.2% and 92.7%, respectively, demonstrating that the nPCR had good accuracy in detecting *A. marginale*, and would be a reliable test for veterinarians to choose the correct treatment for this agent [34].

Despite identifying buffaloes infected with *Babesia* spp., confirmatory evidence for infestation with the vector tick *R. microplus* and the transmission capacity of *Babesia* spp. in water buffaloes has not yet been completely elucidated in Mexico. In a study conducted in Cuba, it was found that *R. microplus* infestation is similar in intensity and frequency in cattle and water buffaloes, also suggesting that the transovarial transmission of *Babesia* spp. in ticks fed on infected buffaloes can be accomplished [35].

Even though genomic DNA from *Babesia* spp. and *A. marginale* was detected, the water buffaloes in this study did not comingle in similar epidemiological conditions with cattle, as was mentioned in other studies [35,36]. The molecular detection of *B. bovis* and *B. bigemina* in water buffaloes has not only been previously reported in Mexico [36], but has also been described in other countries, such as Thailand, Cuba, Sri Lanka, Brazil, and Egypt [12,13,20,21,26,37,38].

In our study, the prevalence of *B. bovis* was higher than that of *B. bigemina* in both water buffalo PU in Veracruz and Tabasco, contrasting with that shown in other studies, where the species with the highest prevalence was *B. bigemina* [35,36]. Recent studies showed higher prevalence rates of *A. marginale* in cattle, as compared to those obtained in water buffaloes [39,40]. In this study, the prevalence rates of *A. marginale* were the lowest in both PU in Veracruz and Tabasco, which could indicate that buffaloes have the immunological ability to control the level of rickettsemia and reduce the infection of this hemoparasite, as previously suggested [14,40].

Mixed infection with the three hemoparasite species was not detected in any of the buffalo samples of our study, as reported in other studies [37,40]. When analyzed in pairs, the mixed infection with *B. bovis* and *B. bigemina* detected in this study was 6.4%, which is considered high when compared to that described in the Philippines [41], but lower when compared to that described in Cuba [13], where the mixed infection reported in water buffaloes was 1% and 20%, respectively. This confirms that this type of infection is not only common in cattle, but also in water buffaloes [40,41,42,43].

The sequence identity for the water buffaloes’ samples in our study was confirmed by comparing the nucleotide sequences with the sequences of *B. bovis*, *B. bigemina*, and *A. marginale*, available in the GenBank database of the NCBI. The homology search using the BLASTn program revealed that the sequences of the gene coding for the RAP-1 protein of *B. bovis* identified in this study shared a sequence identity that ranged from 99.2 to 100% with sequences of *B. bovis* isolates derived from cattle in Brazil (Access No. FJ588009.1), China (KT312806.1), Cuba (MN056340.1), Philippines (LC006978.1), and Portugal (FJ901342.1), but more importantly, with sequences of *B. bovis* isolates derived from buffaloes in Cuba (Access No.JF279443.1) and Sri Lanka (AB845432.1). On the other hand, the highest sequence identity for the *B. bigemina Spe*I-*Ava*I fragment was recorded, with three of the sequences deposited in the GenBank database, and which were identified in Mexican (S45366.1) and Australian isolates (XM_012915429.1 and LK055252.1). The *A. marginale* MSP5 sequences obtained in this study showed up to 99% sequence identity with the isolate sequences published in Brazil (CP023731.1), Egypt (KU042081.1), the Philippines (AB704328.1), and Sri. Lanka (LC467711.1).

The results of this study confirm that water buffalo, as cattle, are also carriers of tick-borne hemoparasites, suggesting that they probably have an important role in the epidemiology of bovine babesiosis in Mexico.

## Figures and Tables

**Table 1:** Primer sequences used in the PCR assays.

Species	Assay	Primer Sequences (5′ > 3′)	Product Size (bp)	Reference
*B. bovis*	PCR	CGAGGAAGGAACTACCGATG	354	[16]
		GGAGCTTCAACGTACGAGGT		
	nPCR	TGGCTACCATGAACTACAAGACTTA	275	[16]
		GAGCAGAACCTTCTTCACCAT		
*B. bigemina*	PCR	CATCTAATTTCTCTCCATACCCC	278	[17]
		CCTCGGCTTCAACTCTGATGCC		
	nPCR	CGCAAGCCCAGCACGCCCCGGT	170	[17]
		CCGACCTGGATAGGCTGTGATG		
*A. marginale*	PCR	ACCTTCTGCTGTTCGTTGC	628	[18]
		TGTACCACTGCCATGCTTAAG		
	nPCR	CATAGCCTCCGCGTCTTT	425	[18]
		CTTAAACAGCTCCTCGCCTT		

**Table 2:** nPCR detection of *B. bovis*, *B. bigemina*, and *A. marginale* in water buffaloes from Mexico.

*Production Unit*	*B. bovis*		*B. bigemina*		*A. marginale*	
	+	%	+	%	+	%
Veracruz 1, *n* = 61	23	(37.7)	6	(9.8)	1	(1.6)
Veracruz 2, *n* = 63	13	(20.6)	7	(11.1)	4	(6.3)
Tabasco 1, *n* = 58	35	(60.3)	8	(13.7)	0	(0)
Tabasco 2, *n* = 58	21	(41.1)	12	(23.5)	1	(1.9)
Total = 233	92	(39.5)	33	(14.2)	6	(2.6)

**Table 3:** Sequence identities of DNA samples from water buffaloes in Mexico, analyzed using BLASTn.

Sample ID	Parasite Sample Source	Accession Number (GenBank)	Highest DNA Sequence Identity (%)	Accession Number (GenBank)
	***B. bovis***			
+Control	Bovine blood	OP174624	275/275 (100)	AF030056.2
4.1	Veracruz	ON862140	273/275 (99)	AF030056.2
17.1	“	ON862141	274/275 (99)	JF279443.1
105.1	“	ON862142	275/275 (100)	AB845432.1
33.1	“	ON862143	275/275 (100)	AB845432.1
50.5	“	ON862144	275/275 (100)	AB845432.1
83.5	“	ON862145	275/275 (100)	AB845432.1
25.1	Tabasco	ON862146	274/275 (99)	JF279443.1
28.3	“	ON862147	274/275 (99)	AB845432.1
33.1	“	ON862148	274/275 (99)	JF279443.1
62.1	“	ON862149	275/275 (100)	AB845432.1
75.1	“	ON862150	275/275 (100)	JF279443.1
94.2	“	ON862151	275/275 (100)	AB845432.1
	***B. bigemina***			
+Control	Bovine blood	OP174630	171/171 (100)	S45366.1
57.1	Veracruz	ON862152	168/171 (98)	S45366.1
68.1	“	ON862153	168/171 (98)	S45366.1
73.1	“	ON862154	168/171 (98)	S45366.1
30.1	“	ON862155	165/170 (97)	S45366.1
100.1	“	ON862156	168/171 (98)	S45366.1
6.1	Tabasco	ON862157	165/171 (96)	S45366.1
23.1	“	ON862158	168/173 (97)	S45366.1
27.1	“	ON862159	168/171 (98)	S45366.1
71.1	“	ON862160	167/171 (98)	S45366.1
75.1	“	ON862161	80/95 (88)	FJ939724.1
88.1	“	ON862162	167/171 (98)	S45366.1
	***A. marginale***			
+Control	Bovine blood	OP174628	425/425 (100)	LC467711.1
218.3	Veracruz	ON862163	408/414 (99)	LC467711.1
236.120	“	ON862164	415/423 (98)	LC467711.1
220.117	“	ON862165	423/426 (99)	LC467711.1
235.32	“	ON862166	303/314 (96)	LC467711.1

## Data Availability

DNA sequences were deposited in GenBank; accession numbers ON862140-ON862151 and OP174624 for *B. bovis*, ON862152-ON862162 and OP174630 for *B. bigemina*, and ON862163-ON862166 and OP174628 for the *A. marginale* isolate’s DNA.
